# Genomic stress in diseases stemming from defects in the second brain

**DOI:** 10.1111/nmo.14860

**Published:** 2024-07-14

**Authors:** Lobke Marie M. Mombeek, Werend Boesmans, David M. Wilson

**Affiliations:** ^1^ Faculty of Medicine and Life Sciences, Biomedical Research Institute Hasselt University Diepenbeek Belgium; ^2^ Department of Pathology, GROW Research Institute for Oncology and Reproduction Maastricht University Medical Center Maastricht The Netherlands

**Keywords:** DNA damage, DNA repair, enteric nervous system, enteric neuropathies, gastrointestinal motility, genomic instability

## Abstract

This review discusses the less‐explored realm of DNA damage and repair within the enteric nervous system (ENS), often referred to as the “second brain.” While the central nervous system has been extensively studied for its DNA repair mechanisms and associated neuropathologies, the ENS, which can autonomously coordinate gastrointestinal function, experiences unique challenges and vulnerabilities related to its genome integrity. The susceptibility of the ENS to DNA damage is exacerbated by its limited protective barriers, resulting in not only endogenous genotoxic exposures, such as oxidative stress, but also exogenous threats, such as ingested environmental contaminants, local inflammatory responses, and gut dysbiosis. Here, we discuss the evidence for DNA repair defects in enteric neuropathies, most notably, the reported relationship between inherited mutations in *RAD21* and *LIG3* with chronic intestinal pseudo‐obstruction and mitochondrial gastrointestinal encephalomyopathy disorders, respectively. We also introduce the lesser‐recognized gastrointestinal complications in DNA repair syndromes, including conditions like Cockayne syndrome. The review concludes by pointing out the potential role of DNA repair defects in not only congenital disorders but also aging‐related gut dysfunction, as well as the crucial need for further research to establish direct causal links between DNA damage accumulation and ENS‐specific pathologic phenotypes.

## Graphical abstract

The enteric nervous system, a.k.a., second brain, is subject to a range of genotoxic exposures that include intrinsic oxidative stress and extrinsic agents associated with ingested foods, inflammation, and dysbiosis. Resulting DNA damage, if left unrepaired, can induce cell dysfunction or death, resulting in gut abnormalities and enteric neuropathy.

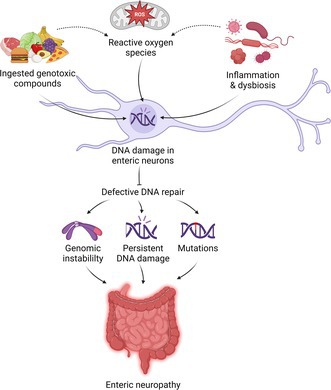

**Key points**

- The enteric nervous system (ENS) is uniquely vulnerable to DNA damage due to its limited protective barriers and exposure to both endogenous factors like reactive oxygen species and exogenous threats such as environmental contaminants and gut dysbiosis.
- Mutations in DNA repair genes such as *RAD21* and *LIG3* are linked to severe enteric neuropathies, including chronic intestinal pseudo‐obstruction and mitochondrial gastrointestinal encephalomyopathy, respectively.
- DNA repair syndromes like Cockayne syndrome likely involve significant gastrointestinal issues, consistent with a crucial role for DNA repair mechanisms in maintaining ENS cell integrity and preventing gut dysmotility.
- Future research is needed to address the many gaps in knowledge regarding the role of DNA damage and DNA repair in enteric neuropathies and the involvement of gastrointestinal tract dysfunction in DNA repair syndromes.

**Abbreviations**
ATP: adenosine triphosphate
BER: base excision repair
CIPO: chronic intestinal pseudo‐obstruction
CNS: central nervous system
DSBR: double‐strand break repair
ENCCs: enteric neural crest‐derived cells
ENS: enteric nervous system
ERCC1: excision repair cross‐complementation group 1
GI: gastrointestinal
HR: homologous replication
HSCR: Hirschsprung Disease
HuC/D: neural Hu proteins C and D
LCL: lymphoblastoid cell lines
LIG3: DNA ligase 3
MMR: mismatch repair
MO: morpholino
mtDNA: mitochondrial DNA
NAD: nicotinamide adenine dinucleotide
NC: neural crest
NER: nucleotide excision repair
NHEJ: non‐homologous end‐joining
NR: nicotinamide riboside
pre‐ENCCs: pre‐enteric neural crest‐derived cells
RAD21: radiation repair gene in yeast
RER: ribonucleotide excision repair
ROS: reactive oxygen species
RUNX1: runt‐related transcription factor 1
WES: whole exome sequencing
XRCC1: x‐ray repair cross‐complementing protein 1

## 1 INTRODUCTION

In mammals, DNA molecules serve as repositories of information crucial for guiding organism development, structural organization, and overall resilience and viability. However, these molecules are susceptible to significant damage from both external and internal factors.^1^ Exogenous factors, such as ultraviolet rays from sunlight and environmental chemicals in the air, food, and water, can alter DNA composition and integrity.^2^ Additionally, endogenous molecules like reactive oxygen species (ROS), which are generated by normal mitochondrial respiration during ATP production, can react with DNA and create unwanted modifications.^3^ When including spontaneous hydrolytic decay, it is estimated that the mammalian genome experiences approximately 10^5^ DNA alterations per day solely from endogenous factors.^4^ These unavoidable changes encompass various changes in nucleotide composition, including depurination (base loss), deamination, alkylation, and oxidation. The different forms of DNA damage span bulky and non‐bulky base adducts, abasic sites, single‐ and double‐strand breaks, interstrand crosslinks, and DNA‐protein adducts.^5^

Given the inevitability of DNA damage, cells have evolved intricate repair systems to address and repair lesions that have the potential to induce genomic instability or disrupt essential processes like transcription or replication. Such adverse molecular outcomes, in turn, may lead to cellular transformation, death, senescence, or other disease‐related endpoints.^6^ The primary DNA damage repair mechanisms include mismatch repair (MMR), which corrects errors introduced during DNA replication; ribonucleotide excision repair (RER), which copes with mis‐inserted ribonucleotides in the genome; nucleotide excision repair (NER), the principal process for eliminating helix‐distorting or “bulky” lesions; base excision repair (BER), the main pathway for resolving oxidative or spontaneous hydrolytic DNA damage; and double‐strand break repair (DSBR), consisting of two mechanisms—non‐homologous end‐joining (NHEJ) and homologous replication (HR)—with the former being error‐prone and the latter highly accurate.^7^ For comprehensive figures detailing all DNA repair mechanisms, refer to the illustrations provided in the following review published in 2022.^8^ The relative contributions of the different DNA repair mechanisms can vary based on the cell's characteristics, such as whether it is dividing or non‐dividing (i.e., terminally differentiated). However, the specific intricacies of this repair variation remain poorly understood for most cell types.^9^ Underscoring the pivotal role of DNA repair pathways in averting undesirable consequences of persistent DNA lesions, both inherited and sporadic mutations in DNA damage repair components underlie a spectrum of clinical manifestations, including cancer, neurological disease, and premature aging.^10^

The enteric nervous system (ENS), often colloquially referred to as the “second brain,” is comprised of enteric neurons and glia primarily derived from the vagal neural crest (NC). As the largest component of the peripheral nervous system, the ENS autonomously coordinates gastrointestinal (GI) function, largely independent of central nervous system (CNS) input. Structurally, it is organized into myenteric and submucosal plexus ganglia, which house diverse neuronal subtypes, including intrinsic primary afferent neurons, interneurons, and motor neurons.^11^ These elements form dedicated circuits crucial for controlling gut functions such as peristalsis and mucosal secretion. Enteric glia, which are present within both the myenteric and submucosal plexus, as well as outside of ENS ganglia, constitute a significant and diverse cell population interacting with enteric neurons throughout the GI tract.^12^ Moreover, enteric glial cells contribute to maintaining GI homeostasis by supporting intestinal barrier function and participating in intestinal immune responses and repair processes.^13^, ^14^ Disruption of the complex neuronal‐glial networks within the ENS can lead to a class of disorders referred to as enteric neuropathies.

The spectrum of enteric neuropathies arising from defects in ENS formation or function can be categorized into developmental neuropathies, acquired neuropathies, neuropathies associated with other diseases, and drug‐induced neuropathies.^15^ One notable example is Hirschsprung disease (HSCR), where the ENS fails to develop in the distal intestine, leading to the absence of propulsive action in the aganglionic region and intestinal obstruction, necessitating surgical intervention in affected newborns.^16^ Similar complications can also arise in adults, resulting in colorectal propulsion failure and the development of megacolon, an abnormal, often toxic, dilation, or hypertrophy of the colon, as observed in Chagas disease caused by *Trypanosoma cruzi* infection.^17^ This pathological progression yields a phenotype similar to that seen in HSCR infants, underlining the critical role of the ENS for the quality of life and survival at any age. The severe consequences of these congenital and sporadic enteric neuropathies highlight the importance of understanding their underlying genetic and molecular causes and identifying the associated opportunities for therapeutic intervention.^18^

## 2 GENOME MAINTENANCE IN THE ENS

### 2.1 Susceptibility of the ENS to DNA damage

Although the DNA repair mechanisms and associated neuropathologies of the CNS have been described in comparatively great detail,^8^ relatively little is known about how genome stability is maintained in the ENS. Furthermore, it is unclear what happens when genome maintenance systems of the ENS are defective. Notably, the ENS lacks the protective confines of the blood–brain barrier or the skeletal structures surrounding the brain and spinal cord. The absence of physical shielding, therefore, renders the ENS susceptible to many challenges, not only from endogenous DNA‐damaging processes, like oxidative stress linked to mitochondrial dysfunction,^19^ but also from external genotoxic threats associated with gut metabolites, inflammatory molecules, pathogens, and environmental contaminants. The close proximity of the ENS to our biggest interface with the outside world, that is, the intestinal epithelium, makes the ENS vulnerable to everything present in our gut lumen. This is accentuated by the fact that the total surface of the GI tract facing the external environment is ∼100 m^2^, with some estimates up to 400 m^2^, in stark contrast to only 2 m^2^ of skin.^20^ Shifts in the microbiome, stemming from various environmental factors such as those associated with diet or infections,^21^ can compromise the integrity of the intestinal barrier by causing perturbations in the lumen.^22^ This so‐called “leaky gut” condition may result in increased exposure to luminal threats and heightened inflammatory conditions, creating an environment where the ENS experiences an elevated onslaught of oxidative stress and genotoxic challenges. These environmental factors, leading to changes in the microbiota, do not only impact the intestinal barrier but also directly affect the ENS.^23^ A Western diet has already been shown to increase the risk for colorectal cancer by promoting genomic stress in intestinal epithelial cells in rats and mice,^24^ indicating a potential to influence DNA damage responses in ENS cells as well. Aging is another significant factor that affects genomic stress, and Jurk et al. reported that DNA damage levels as measured by γ‐phosphorylated H2A Histone Family Member X immunofluorescence increase in enteric neurons of mice with age,^25^ possibly contributing to age‐related gut dysfunction. Such vulnerabilities of the ENS pose the critical question of how the system's cellular components manage to uphold genomic integrity throughout the organism's lifetime, thereby preventing the onset of pathology.

### 2.2 DNA repair in the ENS

**FIGURE 1:**
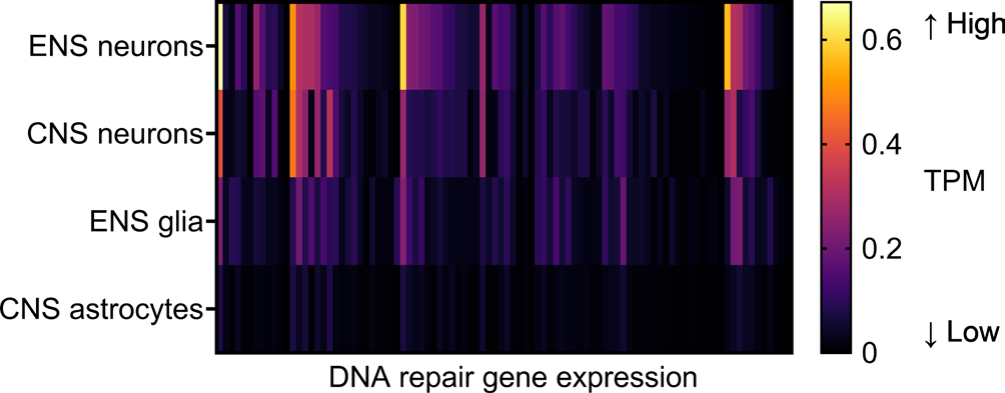
Heatmap of all DNA repair gene expression levels (DNA repair gene list was defined by our laboratory) in ENS neuron, ENS glia, CNS neuron, and CNS glia populations from healthy 12‐ to 30‐day‐old mice. Data were extracted from Linnarson's mouse brain atlas.^26^ Enteric neurons concern populations ENT1–ENT9 of the database, enteric glia concern populations ENTG1–ENTG7, cortical neurons concern populations TEGLU1–TEGLU24, and astrocytes concern populations ACTE1–ACTE2. Gene expression levels per DNA repair gene are reported as transcript per million (TPM) values and were averaged over the respective subpopulations in each class.

Given the range of stressors confronted by the ENS, defining the role of DNA repair in the ENS becomes increasingly relevant. In our laboratory, by leveraging single‐cell RNA sequencing data and mining the Linnarson mouse brain atlas,^26^ we discovered that ENS cells, universally, express higher DNA repair transcript levels than their CNS counterparts (Figure 1). These findings support an essential role for DNA repair in the ENS, and considering its heightened vulnerability to multiple sources of DNA damage, highlights the importance of further research in defining the genome protective mechanisms employed by enteric neurons and glia. In the following section, we describe the emerging evidence indicating that defects in DNA repair can give rise to disorders involving gut motility dysfunction.

### 2.3 DNA repair, DNA damage prevention, and ENS development

NC cells located at the vagal level of the neural tube serve as the primary source of progenitors responsible for generating the ENS. A relatively small pool of pre‐enteric NC‐derived cells (pre‐ENCCs) transforms into enteric NC‐derived cells (ENCCs) upon their migration into the foregut mesenchyme. Proliferating extensively, these ENCCs then migrate along the gut in a rostral‐to‐caudal fashion for timely organ colonization.^27^ Several studies in chick embryos demonstrate that a decrease in the pool of ENS precursors during developmental stages results in a reduction in enteric neuron numbers and varying degrees of hypoganglionosis.^16^, ^28^ Often, the absence of enteric neurons is specific to the distal intestine (colonic aganglionosis),^27^, ^29^ a phenomenon known to occur in HSCR.^30^ Thus, survival of NC‐derived ENS progenitors during the initial phases of intestinal organogenesis is vital. As highlighted earlier, DNA repair genes are integral to the survival of various cell types, including neural progenitors,^31^ and the lack or malfunction of DNA repair mechanisms in ENS progenitors, therefore, would likely lead to impaired ENS development.

#### 2.3.1 Geminin

**FIGURE 2:**
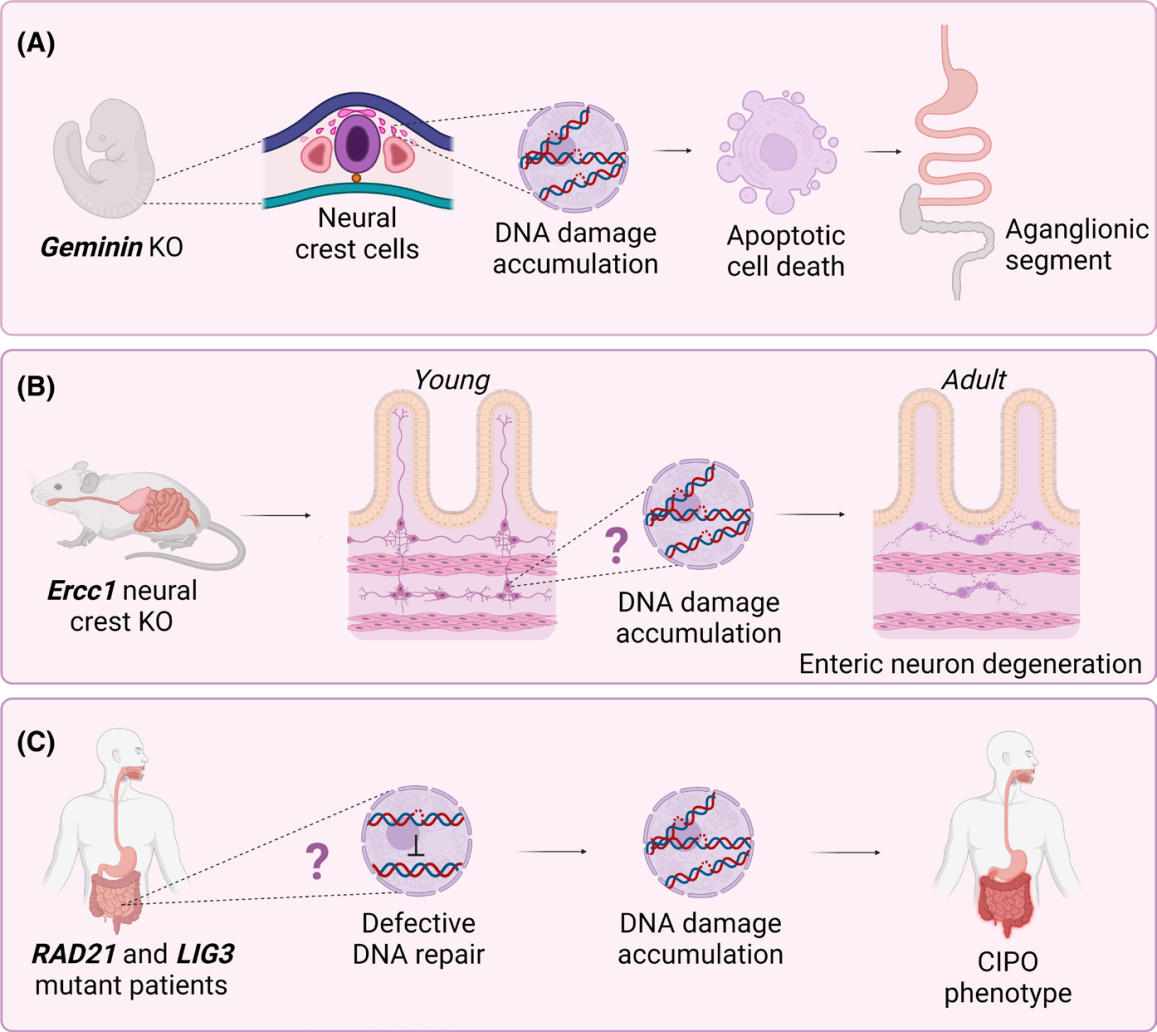
Different DNA repair gene mutations give rise to different phenotypes. (A) KO of Geminin in neural crest‐derived cells has stage‐dependent effects on gut colonization, leading to different degrees of aganglionosis associated with the accumulation of DNA damage. (B) Patients suffering from homozygous RAD21 and LIG3 mutations present with chronic intestinal pseudo‐obstruction (CIPO) as their main clinical feature, which is likely caused by DNA damage accumulating in enteric neurons. (C) Ercc1 knockout (KO) in neural crest cells leads to enteric neuronal degeneration during adulthood, which is reminiscent of late‐onset Hirschsprung disease (HSCR). We hypothesize that these neurons undergo apoptosis due to the accumulation of DNA damage.

Geminin is a cell‐cycle‐dependent protein expressed in all mitotic cells and has been associated with preserving DNA integrity and playing a role in the self‐renewal and commitment of multi‐lineage progenitors.^32^ Considering the crucial importance of both self‐renewal and differentiation for the generation of ENS lineages, Stathopoulou et al. investigated the impact of Geminin in influencing the capacity of ENCCs to undergo proliferation and differentiate into both neurons and glial cells (Figure 2A).^33^ Notably, conditional Geminin inactivation in early‐stage NC cells induces intestinal aganglionosis due to impaired self‐renewal and heightened apoptosis of ENCCs.^33^ In a follow‐up study, Konstantinidou et al. investigated the role of Geminin in the survival of ENS progenitors at additional developmental stages.^34^ They found that deletion of Geminin in early ENS progenitors, prior to foregut invasion, triggers cell‐autonomous activation of the DNA damage response and p53‐dependent apoptosis, resulting in severe intestinal aganglionosis. However, removing Geminin shortly after ENS progenitors invade the embryonic gut does not adversely impact their survival, migration, commitment, or differentiation. Considering the stage‐dependent resistance of ENS progenitors to genotoxic stress that is likely dictated by microenvironmental cues within the embryonic gut, it would be interesting to assess DNA repair capacity in adult ENS cells, as one might speculate that decreases in DNA repair efficiency with age could contribute to GI disorders later in life (i.e., in acquired diseases).

### 2.4 DNA repair defects and enteric neuropathies

While the significance of maintaining genomic integrity likely goes beyond ensuring the survival of ENS progenitors, the evidence thus far for specific DNA repair genes to secure ENS formation and function is centered on the following DNA repair genes: Excision Repair Cross‐Complementation Group 1 (Ercc1), RAD21 (radiation repair gene in yeast), and DNA Ligase 3 (LIG3).

#### 2.4.1 Ercc1

The ERCC1 protein, when in complex with ERCC4 (a.k.a., XPF), forms an endonuclease crucial for NER and other DNA repair (recombination) mechanisms.^35^ Defects in NER give rise to xeroderma pigmentosum, a disorder characterized by extreme UV‐irradiation sensitivity, increased cancer risk (particularly following sun exposure), and neurological abnormalities. With the goal of generating a skin‐specific model for defects in NER, Selfridge et al. established NC‐specific *Ercc1* knockout (KO) mice using two different Cre‐driver lines, Tyr::CreA and Tyr::CreB. Up to the age of 4 months, these *Ercc1* KO mice appeared identical to their littermate controls while exhibiting the expected UV hypersensitivity.^36^ However, between 4 and 6 months of age, all NC‐specific *Ercc1* KO mice experienced a decline in coat condition, began to lose weight, exhibited reduced mobility, and either succumbed rapidly or required culling. Upon further inspection, the authors discovered that both Tyr::CreA‐ and Tyr::CreB‐driver lines resulted in the deletion of *Ercc1* not only in the melanocytes but also in other NC‐derived tissues, including the ENS (Figure 2B). Notably, acetylcholinesterase histochemistry in colons from 118‐day‐old Ercc1‐deficient animals uncovered a severely impaired enteric neuronal network compared to littermate controls lacking Cre expression. Post‐mortem analysis revealed signs of colon distension and fecal impaction; in certain instances, this condition was segmental and limited to the distal colon, whereas in other cases, the entire cecum and colon were affected, a phenotype reminiscent of late‐onset HSCR.^37^ Altogether, this study indicates that degeneration of ENS networks in the large intestine of DNA repair‐deficient mice results in colonic obstruction. Although the paper did not provide a direct causality between DNA damage accumulation and enteric neuronal loss, the results suggest that enteric neurons undergo degeneration due to the buildup of endogenous DNA damage, emphasizing the importance of genome maintenance in gut‐intrinsic neural networks.

#### 2.4.2 RAD21

Chronic intestinal pseudo‐obstruction (CIPO) is characterized by defective intestinal peristalsis, resembling a sub‐occlusive disease without mechanical obstructions.^38^ The lack of understanding regarding specific genetic alterations and molecular mechanisms in most CIPO cases hampers progress in therapeutic options. In prior investigations, Deglincerti mapped a locus within a large consanguineous family that exhibited an autosomal recessive form of CIPO,^39^ for which the clinical phenotype was described by Mungan.^40^ In the affected family members, CIPO was the predominant clinical feature, accompanied by megaduodenum, long‐segment Barrett esophagus, and varying degrees of cardiac abnormalities. Integrating the available mapping data with whole‐exome sequencing (WES), Bonora et al. identified *RAD21* as the causal locus in these patients (Figure 2C).^41^ *RAD21* encodes a protein that operates as a structural component within the highly conserved cohesin complex, which consists of RAD21, SMC1A, SMC3, and SCC3 proteins. This complex plays a key role in sister chromatid cohesion, an event that is essential for accurate chromosome segregation, post‐replicative DNA DSBR, and preventing inappropriate recombination between repetitive genomic regions.^42^

Considering that RAD21 was identified in a forward genetic screen in zebrafish as a main positive regulator of runt‐related transcription factor 1 (RUNX1),^43^ the impact of mutated *RAD21* on *RUNX1* gene expression was explored. RUNX proteins constitute a family of transcriptional regulators playing crucial roles in multiple biological processes, including cell proliferation, apoptosis, differentiation, and cell fate determination, particularly during embryonic development.^44^ Lymphoblastoid cell lines (LCL) prepared from the *RAD21* CIPO‐affected individuals exhibited a significant reduction in *RUNX1* transcription compared to controls. Using zebrafish harboring a *rad21* mutation, *runx1* expression was either partially or completely absent in *rad21a* morphants, aligning with the human LCL results.^43^ Injection of a morpholino (MO) with human wild‐type *RAD21* mRNA restored *runx1* expression in the mutant zebrafish, whereas injection of a MO with mRNA encoding the patient mutation failed to rescue *runx1* expression, consistent with a loss‐of‐function mutation. In line with the CIPO patient phenotype, delayed food transit along the gut was observed in *rad21a* morphant zebrafish. Additionally, quantification of enteric neurons using antibody staining for neural Hu proteins C and D (HuC/D) revealed a depletion of enteric neurons in mutant zebrafish, indicating a neurogenic origin for the observed motility defects. In a follow‐up study, Bianco et al. demonstrated that RAD21 is highly expressed in cholinergic neurons but not in nitrergic neurons of the myenteric plexus of the small intestine in both humans and mice.^45^ These observations, along with the understanding that *RAD21* mutations are present in certain cases of CIPO, suggest that the subset of cholinergic enteric neurons could be the main pathogenic target in these patients.

#### 2.4.3 LIG3

The human genome encodes three distinct DNA ligases (I, III, and IV). While all DNA ligases are present in the nucleus, only ligase III (LIG3) exists as a mitochondrial‐targeted form, arising via alternative splicing. In the nucleus, LIG3 normally interacts with X‐ray repair cross‐complementing protein 1 (XRCC1) to preserve DNA integrity via the BER pathway; yet when defective, the other DNA ligases can generally compensate for its absence. However, in the mitochondria, LIG3 takes on a critical role, serving as the sole ligase for mitochondrial DNA (mtDNA) replication and repair.^46^ Consistently, cellular lethality associated with a *LIG3* null mutation can be ameliorated by directing another DNA ligase to the mitochondria in human cell lines.^47^ Hence, diminished LIG3 activity is anticipated to impact mitochondrial health, potentially giving rise to diseases associated with mitochondrial dysfunction.

Bonora et al. reported a newly identified mitochondrial GI encephalomyopathy resulting from biallelic variants in *LIG3* in three different families (Figure 2C).^48^ This syndrome is primarily characterized by profound gut dysmotility, specifically CIPO. Interestingly, symptom severity varied largely between affected individuals within and between different families. Mitochondrial dysfunction was detected in skeletal muscle biopsies of patients from all three families, together with compound heterozygous variants in the *LIG3* gene. These variants were shared among the affected siblings and inherited from their respective heterozygous healthy parents. Ligation assays revealed that mitochondrial DNA ligase activity in *LIG3* mutant patients was hampered, which led to mtDNA depletion. To verify the role of *LIG3* mutations in inducing the observed clinical phenotypes, the research team explored the impact of LIG3 defects on intestinal neuromuscular characteristics in a zebrafish model. A substantial proportion of *lig3* morphants and KO zebrafish exhibited aberrant gut peristalsis, indicating that impaired *lig3* function results in GI abnormalities.

### 2.5 GI symptoms in patients suffering from DNA repair deficiency syndromes

There is a collection of well‐described DNA repair deficiency syndromes, such as xeroderma pigmentosum, Cockayne syndrome, or ataxia‐telangiectasia,^49^ that harbor pathogenic mutations in specific DNA repair genes. These syndromes, which involve defects in different DNA repair pathways, are associated with various pronounced clinical manifestations, namely cancer predisposition and brain neurodegeneration. However, one historically overlooked symptom present in these syndromes appears to be GI tract issues, seemingly supporting the idea that DNA repair mechanisms play a crucial role in protecting cells responsible for maintaining GI homeostasis. For example, a 3‐year‐old boy with Cockayne syndrome presented with severe malnutrition due to persistent vomiting and diarrhea that did not improve with standard interventions.^50^ Manometric studies revealed abnormal intestinal motility patterns, which responded positively to intravenous metoclopramide, suggesting an association between Cockayne syndrome and intestinal dysmotility and indicating that the neurodegenerative process also affects the intestines. Additionally, post‐mortem analysis of Cockayne patients' bowels revealed abnormalities in the myenteric plexus.^51^ These clinical and pathological features potentially underscore a highly underappreciated role of DNA repair pathways in maintaining the cellular integrity of ENS cells. Moreover, the fact that mutations in *LIG3* and *RAD21* can give rise to clinical phenotypes that predominantly manifest as enteric neuropathies indicate that nervous system‐specific defects can emerge as a GI disorder and/or that certain genome maintenance mechanisms have greater importance in the ENS than in other parts of the nervous system. Finally, as many DNA repair disorders exhibit complications related to malnutrition, poor growth, or broader systemic complications, it seems imperative that ENS abnormalities in these syndromes be investigated more thoroughly. These investigations may, in turn, uncover novel ENS targets for therapeutic intervention in DNA repair deficiency syndromes.

## 3 CONCLUSION

In closing, our review highlights the intricate contribution of genomic stress in neuropathology affecting GI tract structure/function. It also points to the importance of future research in addressing the numerous gaps in knowledge related to DNA damage and repair in enteric neuropathies and gut dysmotility in DNA repair syndromes. Looking to the future,we can start to explore therapies that target DNA repair for the treatment of GI dysmotility disorders. It should be noted that historically, DNA repair‐specific approaches have focused on developing inhibitors for cancer treatment, with little emphasis on the design of activators that could promote general health and prevent disease. One such example gaining traction is the use of nicotinamide adenine dinucleotide (NAD) precursors, which support poly(ADP‐Ribose) polymerase activity essential for efficient DNA strand break repair. NAD precursors, such as nicotinamide riboside (NR), have been reported to shift the gut microbiome, with NR‐conditioned microbiota transplantation reproducing some effects of NR in mice on a high‐fat diet.^52^ Additionally, strategies involving antioxidant supplementation to prevent DNA damage could have therapeutic potential for enteric neuropathies and gut motility disorders. As research progresses in understanding the role of genomic stress in disease pathogenesis and studies advance to develop effective DNA repair system activators, one can begin to envision interventions to promote healthy living and gut function.

## AUTHOR CONTRIBUTIONS

DMW and WB conceptualized the review. LMMM conducted the literature review. LMMM wrote the original draft. DMW and WB reviewed and edited the manuscript. LMMM prepared the visualizations. All authors have read and agreed to the published version of the manuscript.

## CONFLICT OF INTEREST STATEMENT

The authors have declared no conflict of interest.

## Data Availability

The data that support the findings of this study are available from the corresponding author upon reasonable request.
